# Vestibulopathy in Patients Presenting With Ramsay Hunt Syndrome

**DOI:** 10.1097/ONO.0000000000000064

**Published:** 2024-12-13

**Authors:** Erik B. Vanstrum, Eric Smith, Maxwell Weng, Minjung Kim, Ashley Kita

**Affiliations:** 1 Department of Head and Neck Surgery, Los Angeles, California; 2 David Geffen School of Medicine, Los Angeles, California.

**Keywords:** Herpes zoster oticus, Ramsay Hunt syndrome, Vestibulopathy

## Abstract

**Objectives:**

Ramsay Hunt syndrome (RHS) refers to a reactivation of the varicella-zoster virus in the distribution of the facial nerve, but it can involve other cranial nerves as well. In patients with polyneuropathy, the vestibulocochlear nerve is most involved after the facial nerve. The clinical manifestations and long-term vestibular outcomes in these patients remain unclear. This report aims to characterize the clinical course of this rare subset of RHS patients.

**Methods:**

This study is a retrospective case series. The study was conducted in a tertiary care institution. Patients with RHS polyneuropathy presenting with vestibular deficit were reviewed over a 30-year period. Case details including initial presentation, House-Brackmann grade, treatment regimen, vestibular examination and testing, MRI findings, and follow-up time were extracted.

**Results:**

A total of 22 patients were identified. The mean age of diagnosis was 53 years and the average follow-up time was 23 months. Most patients demonstrated complete facial paralysis (88%) upon presentation. Nystagmus was rarely recorded during physical examination (18%, n = 4). For those patients who underwent MRI of the internal auditory canal (n = 17), 59% demonstrated enhancement of the facial nerve, with a notable absence of vestibulocochlear nerve abnormality. Of the patients who underwent vestibular testing, all demonstrated unilateral caloric weakness on videonystagmography (VNG; 66% ± 22%, n = 8).

**Conclusions:**

Patients with RHS polyneuropathy and vestibular complaints do not consistently demonstrate objective vestibular physical examination or imaging findings on presentation. However, VNG consistently demonstrates significant unilateral weakness on caloric stimulation. Most patients in our sample continued to have vestibular complaints at latest follow-up.

In 1907, James Ramsay Hunt first described a triad of symptoms that has since become eponymous, a vesicular rash of the auricle, ipsilateral facial palsy, and auditory symptoms (1). In this report, Hunt outlined the infectious, anatomic, and clinical basis for herpes zoster oticus and described 3 clinical groups of increasing severity. With regards to the most severe form of the syndrome, he described patients who develop “symptoms of Meniere disease” including disturbances of equilibrium, vertigo, nausea, and nystagmus.

Today, the classic hallmarks of Ramsay Hunt syndrome (RHS) are similarly recognized, with more emphasis placed on a triad including otalgia as opposed to vestibular symptoms. While RHS is a rare disease entity, occurring with an incidence estimated at 5 per 100,000 individuals per year (2), there are infrequent presentations of RHS, which include polyneuropathies as Hunt first alluded to in his original work describing cranial nerve (CN) VIII involvement (3). Despite these original observations, the scientific and clinical analysis of RHS with polyneuropathy remains limited to selected reports.

The long-term outcomes of those patients with RHS and vestibular nerve involvement are unclear and understudied, especially with regard to their recovery from vestibular deficit. There are challenges to studying vestibular outcomes in general, and especially in exceptionally rare diseases. Polyneuropathy involving the vestibular nerve is thought to account for only ~10% of RHS infections (4,5). Further, a wide variety of providers treat RHS including primary care providers, emergency physicians, and otolaryngologist. While facial palsy and vesicles are readily apparent on exam, nystagmus and signs of vestibular weakness can be difficult to recognize, and subjective complaints of vertigo can be misconstrued. Further, objective vestibular testing requires specialized equipment and personnel. As such, there are no longitudinal reports on the recovery of patients with RHS and vertigo on presentation. The aim of this report is to evaluate a case series of patients with RHS polyneuropathy of the vestibular nerve and to explore trends in vestibular recovery following infection.

## METHODS

This study qualified for exemption by the Institutional Review Board at the University of California Los Angeles (IRB#23-000095). A retrospective review of patients diagnosed with RHS and who presented with vestibular nerve involvement was performed from the University of California Los Angeles records dated 1990–2022. An initial screen of the medical record utilized a combination of International Classification of Diseases-9/10 codes and key words (Supplemental Material, http://links.lww.com/ONO/A33), yielding 320 patients. A detailed review of these resulted in a final dataset of 22 patients. Inclusion criteria were patients with a diagnosis of RHS and subjective complaints of vertigo, dizziness, or imbalance upon initial presentation. Exclusion criteria included patients without acute symptoms of vertigo/dizziness/imbalance upon presentation, patients with systemic zoster infections, or patients with alternative causes or explanations for vertigo (eg, presence of vestibular schwannoma, history of vestibular migraine, and history of temporal bone resection). Those records that met inclusion and exclusion criteria but did not contain sufficient information were also excluded. Examples of patients with insufficient information include those without follow-up after initial presentation, or whose primary infection was treated at an outside facility and minimal records were available for review. Clinical, demographic, and radiologic data were extracted. A complete list and dataset of extracted variables for each case is included in Supplemental Material, http://links.lww.com/ONO/A33 and may be used by future authors for meta-analysis. In addition, a succinct case report is presented for each patient highlighting vestibular symptoms, examination, treatment, and recovery. Twenty percent of the 320 reviewed cases were diagnosed with RHS, while the others included mention of RHS but were not formally diagnosed, for example, “differential diagnosis includes RHS” or “rule out RHS.”

## RESULTS

We estimate that 243 patients were diagnosed with RHS at our institution within the study period. The final cohort comprised of 22 patients with a mean age of 53 years (Table 1), suggesting about 9% of patients had a concomitant vestibular polyneuropathy. About 55% were female (n = 12). Most patients presented with a complete facial palsy (median House Brackmann [HB], interquartile range; 6, 0). The most common accompanying cochlear symptoms included hearing loss (59%), tinnitus (45%), aural fullness (23%), and hyperacusis (18%). Four patients were noted to have spontaneous nystagmus upon presentation or had nystagmus elicited by exam (18%). All patients were treated with a course of steroids and antivirals, except for one patient that solely received antivirals.

**TABLE 1.** Summary of clinicodemographic information, n = 22 (%, n)

Age (mean years ± SD)	53.3	15.4
Female (%, n)	55%	12
Laterality		
Left (%, n)	45%	10
Presentation		
Initial HB (median, IQR, n = 17)	6	0
Unclear (n)	5	
Cochlear vestibular symptoms (%, n)		
Aural fullness	23%	5
Hearing loss	59%	13
Hyperacusis	18%	4
Tinnitus	45%	10
Vertigo	100%	22
Treatment (n = 20)		
Steroids	95%	19
Antivirals	100%	20
Imaging (%, n)		
MRI IAC	77%	17
Facial nerve enhancement	59%	10
Audiology assessment		
Audiogram (%, n)	45%	10
Audiogram <3 mo from diagnosis (%, n)	36%	8
>10 dB difference in PTA (affected − unaffected side) (%, n)	60%	6
VNG (%, n)	36%	8
Physical therapy (%, n; among those with >6 mo follow-up)	31%	5
Time to latest follow-up, months (median, range)	23	1–204
Persistence of vestibular symptoms at the latest follow-up	86%	19

HB indicates House-Brackmann; IAC, internal auditory canal; IQR, interquartile range; PTA, pure tone average; VNG, videonystagmography.

**FIG. 1.**
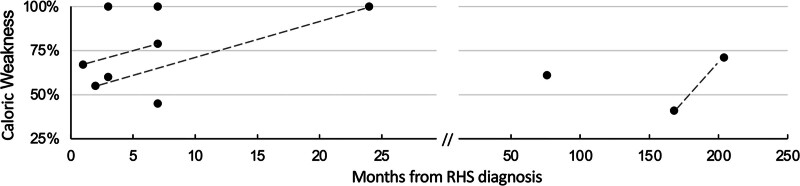
Summary of VNG caloric testing. Of patients who underwent VNG (n = 8), 100% demonstrated ipsilateral vestibular weakness on caloric testing. Three patients underwent repeat VNG (dashed lines). VNG indicates videonystagmography.

Seventeen patients underwent an MRI of the internal auditory canal, and of these patients, 59% (n = 10) demonstrated facial nerve enhancement. About one-third of patients (36%, n = 8) underwent auditory testing within 3 months of diagnosis, and of these patients, 60% (n = 6) demonstrated asymmetric hearing loss of the affected side. About one-third of patients underwent videonystagmography (VNG; 36%, n = 8; Fig. 1), of which 100% demonstrated ipsilateral vestibular weakness on caloric testing. Three patients underwent repeat VNG and demonstrated continued weakness. The median time to latest follow-up was 23 months (range: 1–204 months). Most patients described some degree of persistent vestibular symptoms at latest follow-up (86%, n = 19). For patients with greater than 6 months follow-up (n = 16), a succinct case report was detailed to highlight the initial presentation of the disease and clinical course with a focus on vestibular complaints, examination, and prognosis (Supplemental Material, http://links.lww.com/ONO/A33). Of these patients, 8 (50%) continued to have some degree of facial palsy at latest follow-up with median HB II/VI. The other 8 patients had complete resolution of paralysis. One patient had persistent facial paralysis of HB IV and no patients had complete paralysis. No patients required surgery to address their weakness temporarily or permanently. Fifteen of the 16 patients (94%) described persistent vestibular symptoms at latest follow-up. One patient had persistent cranial X sensory neuropathy diagnosed on functional endoscopic evaluation of swallow, which was attributed to RHS polyneuropathy; no other patients had documented additional neural deficits. Five (31%) of patients underwent vestibular physical therapy (PT).

## DISCUSSION

There is evidence in the literature to counsel patients on their recovery of facial nerve function and hearing loss after an acute RHS infection (4–6), but there is no data on the long-term prognosis of patients with RHS and vestibular deficits. The results of this case series suggest that most patients with acute vertiginous symptoms after RHS infection continue to struggle with peripheral vestibular weakness well after their diagnosis. VNG months and years from initial infection demonstrate peripheral vestibular deficit. While there are limitations inherent to case series chart review, our results nonetheless can educate clinicians on how to counsel patients regarding their recovery from vestibular deficit and inform treatment decision-making by encouraging early vestibular PT intervention.

There are several important observations from this review. First, vestibular symptoms occur at least 1 in 10 patients with RHS but are not often recognized on initial presentation. Numerous patients in our series were accurately diagnosed with RHS, but their subjective complaints of vertigo and dizziness were misconstrued as anxiety. Most often these symptoms are elicited on history rather than physical exam, as few patients report documented spontaneous nystagmus or corrective saccades on head impulse testing. This may be attributed to a knowledge gap among first-line providers who may not recognize polyneuropathy as a rare complication of RHS, or who may not be trained or equipped to pursue vestibular examination, such as performing head thrust testing. When performed by otolaryngologists specialized in vestibular care, 60% of patients with RHS and dizziness had positive head impulse testing (7). Only 2 patients in our sample were noted to have positive head impulse testing findings. Such disparity highlights how patients are evaluated differently when they receive prompt neurotology specialty care and perhaps a lack of awareness of vestibular symptoms occurring in RHS patients.

Second, nearly all the patients in our sample demonstrated complete facial paralysis on RHS presentation, suggesting that vestibular deficit should be queried in those with more severe RHS infections as they may be prone to developing polyneuropathy. Kim et al (8) demonstrate that in a sample of 160 patients with RHS, the incidence of vestibulopathy increased as the severity of facial palsy increased. Similar findings are demonstrated in prior studies, though in these instances testing occurred exclusively shortly after presentation (7,8). Compared to these prior studies, this study benefits from longer follow-up times. Our results suggest that of those describing continued vestibular symptoms, most had improvement in facial paralysis, indeed, half of the patients had complete resolution of facial palsy. In other words, while facial palsy demonstrates clinically meaningful improvement, vestibular complaints persist and are demonstrated by persistent caloric weakness on repeat VNG examination.

Third, most patients with RHS who present with vestibular symptoms have persistent vestibular symptoms months and years after their diagnosis. While this result may be overestimated as patients with resolution of symptoms might not continue to pursue care, it does suggest that increased awareness should be made of the pathological relationship and potential for long-term vestibular consequences after RHS infection. While vestibular examination and VNG can aid in the objective diagnosis of vestibulopathy, our results suggest that recognizing acute vertigo during an initial history by first-line providers is sufficient to provide a vestibular PT referral. Vestibular PT remains the principal treatment for patients with unilateral vestibular hypofunction (9). Indeed, many patients in our sample described subjective improvement in symptoms with vestibular PT. It should be noted that several patients in our sample were prescribed vestibular suppressants (eg, meclizine), which can be effective in mitigating acute symptoms but are not recommended longitudinally because they can suppress central compensation in peripheral hypofunction, hindering recovery.

Less than one-third of patients were prescribed vestibular PT in our cohort, demonstrating significant room for improving clinical care in this patient cohort. An emphasis on prompt treatment with vestibular PT would likely have helped numerous patients in this cohort, some of whom waited years before the diagnosis of a peripheral vestibular lesion. Future studies might evaluate these patients longitudinally with validated scoring measures such as the dizziness handicap index rather than relying on subjective complaints.

Not all patients with RHS demonstrate facial nerve enhancement on MRI, and no patients within our sample demonstrated vestibular nerve enhancement. Isolated vestibular nerve enhancement can be visualized in instances of vestibular neuritis, though a high dose of gadolinium is required (10). The discrepancy in enhancement between the facial and vestibular nerves has been attributed to anatomy, as the facial nerve has a more prominent circumneural vasculature (11). In regards to facial nerve recovery, our sample is consistent with prior studies on RHS without polyneuropathy demonstrating that about half of patients recover completely (12).

Hearing loss and vertigo do not always present simultaneously. For example, in case #21, no hearing loss was demonstrated by an audiogram, but 100% caloric weakness was discovered on VNG. In a series of 186 patients with RHS, of those with normal hearing, only 5% complained of vertigo, compared with 25% of patients who had documented hearing loss (4).

While the strengths of this study include a longitudinal, detailed, case review of a large series of patients with RHS polyneuropathy and vestibular symptoms, there are significant limitations. This series is heterogeneous and therefore rigorous statistical analysis is not feasible. Multiple providers evaluated and treated these patients over a multidecade period. This included a review of clinical notation from emergency medicine, family medicine, internal medicine, otolaryngology, neurotology, neurology, and infectious disease physicians. As such, there was no standard physical examination, treatment, pharmacologic, or clinical workup algorithm. While limiting robust statistical conclusions, our institution’s experience is reflective of a real-world experience, which allowed our review to highlight important lessons in the treatment of vestibulopathy in patients with RHS.

## CONCLUSION

While vestibular insult during RHS infection is rare, its associated vertigo can be debilitating. Clinicians should both ask about and look for signs of vestibular deficit upon initial presentation and perform a full CN examination in patients suspected to have RHS. Individuals with a full facial paresis are at higher risk for the involvement of other CNs. Prompt initiation of steroids and antivirals should occur.

As all patients in our sample who underwent VNG demonstrated a peripheral vestibular deficit on caloric testing, any suspected vertiginous symptoms in RHS are highly suggestive of a measurable vestibular deficit. Vestibular PT should thus be prescribed expeditiously, even in the absence of VNG confirmation. Patients should further be counseled that continued vestibular disturbance can persist for months and years after diagnosis, but also that vestibular PT helps with accommodation.

## FUNDING SOURCES

None declared.

## CONFLICT OF INTEREST STATEMENT

None declared.

## Supplementary Material


