# Supplementary material for: Vestibulopathy in Patients Presenting With Ramsay Hunt Syndrome
Source: Otol Neurotol Open. 2024 Dec 13;4(4):e064. doi: 10.1097/ONO.0000000000000064 (PMC11671088; doi:10.1097/ONO.0000000000000064)
Supplement: Supplementary file 1 [file ono-4-e064-s001.pdf]

*Supplemental Case Reports*

Case 1

A 55-year-old female presented with two-day history of left sided hyperacusis, vertigo with lateral head movement, hearing loss, tinnitus, and House-Brakmann (HB) 6/6 left facial palsy. She was treated with 14 days of antivirals and 6 days of prednisone. At 4 months, she complained of persistent vertigo with head movement. Her neurology examination as notable for a negative Romberg exam and unsteady tandem gait. During the same time frame, the patient was examined by a neurotologist, who noted that the patient had an uncompensated left peripheral vestibular loss. No videonystagmography (VNG) was ordered due to anticipation of improvement over the next several months. At 1 year and 6 months, the patient continued to have vertigo, along with tinnitus. At 1 year 8 months, on neurology examination the patient was noted to ambulate well, but with inability to tandem walk, and had an unsteady Romberg. At 2 years, the patient was noted to have much improved vertigo after evaluation by her primary care physician.

Case 2

A 66-year-old male presented with right-sided aural fullness, tinnitus, vertigo, facial palsy and vesicles of the external auditory canal. They were treated with a course of antivirals and steroids at an outside hospital. At 12 months, the patient was examined by an otolaryngologist and was noted to have normal gait and no spontaneous nystagmus. His mains symptoms included right sided tinnitus, vertigo. Electronystagmography was ordered but not completed and the patient was lost to follow up.

Case 3

A 35-year-old female presented with right HB 6/6 facial palsy, vesicles of the pinna, and vertigo. At 4 years, on examination by a neurologist, her stance and gait were unremarkable, her Romberg was normal. The patient noted continued vertigo with movement. There was concern that her symptoms were precipitated by anxiety, and she was referred to psychiatry without further follow up.

Case 4

A 68-year-old male presented right HB 6/6 facial palsy, right sided hearing loss, vertigo, and vesicles of the right palate He was treated with 10 days of steroids and acyclovir. His MRI was normal. At 6 years, on examination by his primary care provider, he is noted to have residual dizziness since his diagnosis. His audiogram at this time demonstrated no asymmetric hearing. He was referred to evaluation by a neurotologist, which is pending.

Case 5

A 75-year-old female with Sjogren's syndrome initially presented with left facial weakness HB 6/6, vesicles of the EAC, hearing loss, otalgia, dizziness, and pain with loud noises. Four days from symptom onset, she was diagnosed with RHS and treated with one week of Acyclovir 1000mg TID and prednisone 60mg qday. Her vertiginous symptoms were aggravated with head movement and ambulation, causing nausea. She pursued vestibular physical therapy, 2 times per week for 8 weeks, and was noted to progress to functional independence walking in the community and gained the ability to drive, though still with continued poor static and dynamic

balance. She remained at risk for falls particularly with walking while head turning or eyes closed activities. A VNG 2 months later demonstrated 55% caloric paresis on the left side. At 3 months she was evaluated for post-infectious dysphagia by a laryngologist and noted to have laryngeal sensory neuropathy due to RHS, with normal bilateral vocal cord movement. Auditory examination at one month, one year, and three years demonstrated left-sided, stable asymmetric hearing loss. She underwent an MRI IAC at 1 year which demonstrated no vestibulocochlear or facial neural enhancement. At two years from symptom presentation, she was noted to have a strongly positive Romberg and difficulty with tandem walking. A repeat VNG at this time demonstrated 100% caloric weakness on the left.

Case 7

A 74-year-old female presented with right sided HB 6/6 facial palsy, vertigo, and vesicles of the EAC and helix. Her MRI was normal. She was treated with a course of antivirals and steroids. At 3 years, she had continued balance dysfunction but was improving with vestibular therapy.

Case 8

A 48-year-old male presented with left-sided hearing loss, and acute vertigo such that the patient could not stand on his own. Two weeks later, he developed left facial palsy HB 6/6 and vesicles of the auricle. He was treated with 5 days of prednisone and acyclovir. He continued to have persistent vertigo and 7 months later was evaluated by a neurotologist. At this time VNG demonstrated 100% caloric paresis on the left. At 9 months he was evaluated by neurology and on examination was noted to have normal gait but diminished tandem stance with eye closure. Subjectively, he noted that his imbalance had improved but was still apparent with head movement, especially in a darkened setting. He was evaluated three years later and was noted to have continued facial paralysis HB 2/6 along with continued vertigo.

Case 10

A 23-year-old female presented with headache, right-sided facial paralysis HB 6/6, otalgia, and vesicles on the pinna. She was treated with 5 days of acyclovir and no steroids. The patient was lost to follow up and re-presented 17 years later to our hospital system. At this time, she complained of chronic vertigo and further workup was initiated. A VNG demonstrated right-sided caloric weakness of 41% with vestibular compensation. Two years later and 19 years from diagnosis, she continued workup for episodes of vertigo. A repeat VNG showed 71% right caloric paresis, again with absence of spontaneous nystagmus suggesting a compensated right vestibular deficit. Her MRI IAC was noted to be normal without evidence of endolymphatic hydrops – please note that the author’s institution uses a delayed contrast MRI to evaluate hydrops. Zoster titers showed evidence of prior infection but no acute infection; elevated IgG and normal IgM. She is pending further evaluation by a neurotologist.

Case 11

A 38-year-old female who was 28 weeks pregnant with her first child presented with left-sided otalgia, facial paralysis HB 6/6, vesicles on the pinna, and vertigo with head movement. She was treated initially with IV acyclovir and solumedrol, then transitioned to oral acyclovir and prednisone. Her vertigo improved subjectively but was still noted to occur with quick head movements. An audiogram at 3 months showed no hearing deficit. Electromyography indicated a severe axonal lesion of the left facial nerve with involvement of all branches. One year later, she

returned to work as a surgeon, but only part time due to her continued vertigo. 5 years later she had recovery of all facial nerve branches except weakness to the marginal branch, as well as synkinesis of the lower branches. She continued to have dizziness with head movement. At 6 years she had a VNG demonstrating 61% caloric weakness on the left. On physical examination by a neurologist, she was demonstrated to have covert saccades to the left with head impulse testing, sway with closed-eye Romberg, and 180 degrees turn to the left on Fukada step test after 25 steps.

Case 12

A 44-year-old male presented with 1 day of left otalgia, hearing loss and vertigo. Vesicles were noted along the ear and palate. On exam he demonstrated horizontal nystagmus, worse with gaze to the right, and upward gaze with diagonal nystagmus. An MRI demonstrated faint enhancement of the left internal auditory canal. He was prescribed acyclovir and prednisone. At 4 months he was evaluated by physical therapy and noted to have loss of balance and veering to one side with head motion, unsteadiness when walking on uneven surfaces, and loss of balance when attempting to play sports. At 6 months, the patient continued vestibular physical therapy and was noted be improving, but still had difficulty with walking and simultaneous head motion. An updated home exercise program was assigned with faster paced movements including walking and jogging. At 21 months, he was noted to have complete facial nerve function recovery with repeat MRI internal auditory canal showing resolution of previously noted left facial nerve enhancement.

Case 16

A 67-year-old female, otherwise healthy, presented with painful burning lesions of the left palate and left ear canal. About one week later, she developed severe vertigo and was prescribed meclizine. Shortly after, she developed left facial palsy HB 6/6, was dysarthric, and had difficulty swallowing solids. Her workup included an audiogram which showed profound left sensorineural hearing loss. At 1 month, the patient noted severe balance disability and she required assistance with activities of daily living to the degree that an otolaryngologist recommended that the husband take 4 months of family leave from his job to care for her. She underwent vestibular physical therapy and demonstrated interval improvement 6 months later. She was evaluated by neurology at this time and noted to have likely involvement of the 9th, 10th, and possibly 5th cranial nerves. One and a half years later, she complained of continued imbalance when walking and moving her head. She suffered two ground level falls without major injury. On physical examination she was noted to have positive head thrust to the left and difficulty with tandem walking. Seven years later she was evaluated by neurotology and complained of continued imbalance. Further vestibular therapy was recommended. The patient was referred for VNG multiple times but did not complete testing. At last visit her facial palsy was HB 3/6.

Case 17

A 33-year-old female with pulmonary hypertension presented with 5 days of left otalgia and ear swelling, which was treated with bacitracin at an outside hospital. At this time, she presented to the emergency room with left facial palsy HB 6/6, and vertigo, worse with head movement. Her vertigo was attributed to anxiety, and she was administered a benzodiazepine. Her MRI was significant for enhancement of the geniculate ganglion of the left facial nerve. She was admitted

overnight for IV antibiotics and steroids. No vesicles were noted on examination. On discharge she was sent home with prednisone, acyclovir, and Augmentin. After one month she was noted to have tinnitus and an erythematous EAC but no vesicles. At two months she was evaluated by audiology and otolaryngology. Her audiogram showed no hearing deficit. Her otolaryngology exam demonstrated healing wounds within the EAC believed to be consistent with RHS. At three months she was evaluated by a neurologist for continued vertigo. On physical examination she was noted to have normal gait, a negative Romberg, and tandem walk that was sidestepped and difficult. Her VNG showed 60% decreased response to caloric stimulation, suggesting a left compensated peripheral vestibular lesion. At 11 months she had undergone vestibular physical therapy with home exercises and noted subjective improvement of her imbalance. On examination at this time she had a normal Romberg, and normal tandem step. Her facial palsy was noted to be HB 2/6.

<u>Case 18</u>
A 60-year-old female presented with a two-day history of left sided HB 6/6 facial palsy and vertigo. Her MRI was normal. She was treated with 3 weeks of acyclovir and prednisone. At 3 years, the patient was evaluated by neurology for persistent vertigo with movement and tinnitus. On examination, her tandem step was unstable, her Romberg with eyes closed was positive, and head thrust to the left showed corrective saccades indicating a left peripheral vestibular lesion. Her neurologist concluded that it would be highly atypical after an acute event to have persistent vertigo and that anxiety may be complicating the recovery of her left vestibular deficit. His facial palsy had completely resolved.

<u>Case 20</u>
At 33-year-old male presented with 6 days of dizziness, headaches, loss of balance, and incomplete left facial paralysis. On exam he had vesicles along the left concha and anterior helix. He had a positive head thrust test and no spontaneous nystagmus. MRI revealed linear enhancement involving the canicular, labyrinthine, and tympanic segment of the left facial nerve and geniculate ganglion. He was prescribed valacyclovir 1000mg TID and prednisone 60 mg for 7 days. At one month, audiometry showed a mild left sensorineural hearing loss. His VNG showed a 67% caloric weakness on the left. He pursued vestibular physical therapy. After 7 months, his facial paralysis resolved. Repeat VNG showed 79% caloric weakness on the left. At 10 months, the patient felt that the improvement in his symptoms had plateaued. He noted difficulty with gaze fixation when running or playing basketball. His vestibular therapist noted a reliance on stable vision for gait stability and drifting to the left without visual input. At his last visit the plan was to continue vestibular therapy.

<u>Case 21</u>
A 64-year-old male presented with right-sided tinnitus, vertigo, HB 6/6 facial palsy after seven days of symptom onset. An MRI demonstrated enhancement along the distal tip of the superior portion of the right IAC extending into the geniculate ganglion of the right facial nerve. They were treated with a course of acyclovir and prednisone. An initial audiogram demonstrated no significant or symmetric hearing loss. At 3 months, VNG demonstrated 100% right sided caloric weakness. At 14 months, patient noted continued subjective vertiginous episodes and dizziness.

<u>Case 22</u>

A 35-year-old-male presented with vertigo, right HB 6/6 facial palsy, and vesicles within the EAC. MRI demonstrated enhancement of the left facial nerve involving the distal canicular segment, labyrinthine segment, geniculate ganglion, tympanic segment, mastoid segment, and visualized portion in the stylomastoid foramen. He was treated with 7 days of prednisone and 3 weeks of valacyclovir. At 7 months he was found to have continued imbalance and dizziness and was ambulating with a cane. He described worsening symptoms with ambulation and occasional room spinning vertigo that resolves spontaneously. He then underwent VNG demonstrating right sided caloric weakness of 45% and was prescribed vestibular PT.

*Supplemental Material: Search Criteria*

1. Patients with B02. 22 postherpetic neuralgia (ICD 10 code) OR 053.11 postherpetic neuralgia (ICD 9) OR "Ramsay Hunt" OR "Ramsey Hunt" OR "herpes zoster oticus"

**AND**

2. H55 nystagmus (ICD 10) OR 379.50 nystagmus (ICD 9) OR 92547 ENG (CPT) OR 92537 caloric irrigation (CPT) OR 92541 Vestibular Function Tests, With Recording (CPT) Nonspecific abnormal vestibular function studies (794.16) OR 92540 Basic vestibular evaluation OR VNG (CPT 92542) OR R42 Vertigo (ICD 10) OR 780.4 Vertigo (ICD 9)
